# GABAergic mechanisms involved in the prepulse inhibition of auditory evoked cortical responses in humans

**DOI:** 10.1371/journal.pone.0190481

**Published:** 2018-01-03

**Authors:** Koji Inui, Nobuyuki Takeuchi, Shunsuke Sugiyama, Eishi Motomura, Makoto Nishihara

**Affiliations:** 1 Institute for Developmental Research, Aichi Human Service Center, Kasugai, Japan; 2 Department of Integrative Physiology, National Institute for Physiological Sciences, Okazaki, Japan; 3 Depatment of Psychiatry, Aichi Medical University, Nagakute, Japan; 4 Department of Psychiatry, Gifu University Graduate School of Medicine, Gifu, Japan; 5 Department of Neuropsychiatry, Mie University Graduate School of Medicine, Tsu, Japan; 6 Multidisciplinary Pain Center, Aichi Medical University, Nagakute, Japan; Tokai University, JAPAN

## Abstract

Despite their essential roles in signal processing in the brain, the functions of interneurons currently remain unclear in humans. We recently developed a method using the prepulse inhibition of sensory evoked cortical responses for functional measurements of interneurons. When a sensory feature is abruptly changed in a continuous sensory stimulus, change-related cortical responses are recorded using MEG. By inserting a weak change stimulus (prepulse) before the test change stimulus, it is possible to observe the inhibition of the test response. By manipulating the prepulse–test interval (PTI), several peaks appear in inhibition, suggesting the existence of temporally distinct mechanisms. We herein attempted to separate these components through the oral administration of diazepam and baclofen. The test stimulus and prepulse were an abrupt increase in sound pressure in a continuous click train of 10 and 5 dB, respectively. The results obtained showed that the inhibition at PTIs of 10 and 20 ms was significantly greater with diazepam than with the placebo administration, suggesting increased GABA_A_-mediated inhibition. Baclofen decreased inhibition at PTIs of 40 and 50 ms, which may have been due to the activation of GABA_B_ autoreceptors. Therefore, the present study separated at least two inhibitory mechanisms pharmacologically.

## Introduction

The outputs of a neural circuit are influenced by the balance between excitation and inhibition produced by pyramidal cells and interneurons, respectively. Despite their crucial roles in signal processing, the functions of interneurons remain largely unknown in humans mainly due to methodological limitations associated with the observation of their activities. The recording of inhibitory postsynaptic potentials (IPSPs) or currents (IPSCs) in the brain slices of animals enables direct observations of the synaptic events mediated by interneurons via GABA. However, there is currently no method to record IPSPs in their pure form in humans. This recording *in vivo* is also challenging in animals. Since many diseases, such as schizophrenia [1–4] and developmental disorders [5,6], have been suggested to have deficits in inhibitory functions or excitation/inhibition imbalances [7,8], non-invasive methods for functional measurements of the inhibitory system are needed.

We recently developed a method for this purpose using the prepulse inhibition (PPI) of auditory evoked cortical responses [9–11]. It appears to serve as a tool to record IPSPs indirectly. When a sound feature is abruptly changed in a continuous sound, change-specific cerebral responses are evoked that are clearly recorded by electroencephalograms [12,13] or magnetoencephalograms (MEG)[14]. Change-related brain activity is used as a test response that reflects postsynaptic events in the change-detecting circuit in the auditory cortex [11]. Similar to standard paradigms for the PPI of startle reflexes, a weak change in a sound feature is inserted before the test stimulus, which results in the suppression of the test response despite the weak change stimulus (prepulse) itself evoking no or only a weak response [9,11]. This paradigm is advantageous because a weak prepulse may be precisely and repeatedly delivered. Change-related brain activity may be elicited by a change in any of the sound dimensions, i.e., intensity, location, and frequency [12], and a brief preceding sound with any feature change may be a prepulse. In our recent studies, the degree of PPI depended on the prepulse–test interval (PTI)[11] as well as the magnitude of the change in the prepulse [9]. When the magnitude of PPI was plotted against the PTI, the PPI curve showed several peaks at different PTIs: 10–30, 50–60, and 600 ms [11]. These findings suggest that the suppression of the test response reflects an active inhibitory process because the prepulse only evoked a weak response if present, and that the PPI curve plotted against the PTI appears to reflect the time course of IPSPs because the inhibitory process was not significantly confounded by changes in pyramidal cell-pyramidal cell transmission [11]. Given that the PPI curve against the PTI reflects the time course of IPSPs, several peaks at different PTIs suggests temporally distinct several mechanisms involved in this suppression.

In the present study, we attempted to separate these inhibitory mechanisms pharmacologically using diazepam and baclofen, a positive modulator of the GABA_A_ receptor and agonist of the GABA_B_ receptor, respectively. The results obtained showed early and late inhibition sensitive to diazepam and baclofen, respectively, confirming that the GABAergic system is involved in shaping the PPI of auditory evoked cortical responses and also indicating that this measure may be a clinical tool for functional assessments of the inhibitory system mediated by GABA_A_ and GABA_B_ receptors.

## Methods

This study was approved in advance by the Ethics Committee of the National Institute for Physiological Sciences, Okazaki, Japan and conducted in accordance with the Declaration of Helsinki. Written consent was obtained from all subjects. The experiment was performed on ten (three females and seven males) healthy volunteers, aged 20–52 (33.2 ± 10.9) years. They were asked to refrain from alcohol, caffeine, and smoking for at least 12 hours prior to the experiment. None of the subjects had any history of mental or neurological disorders or substance abuse in the last two years. They were free of medication at testing. They had a hearing threshold lower than 30 dB at 1000 Hz, as assessed by an audiometer (AA-71, Rion, Tokyo, Japan).

### Auditory stimuli

**Fig 1:**
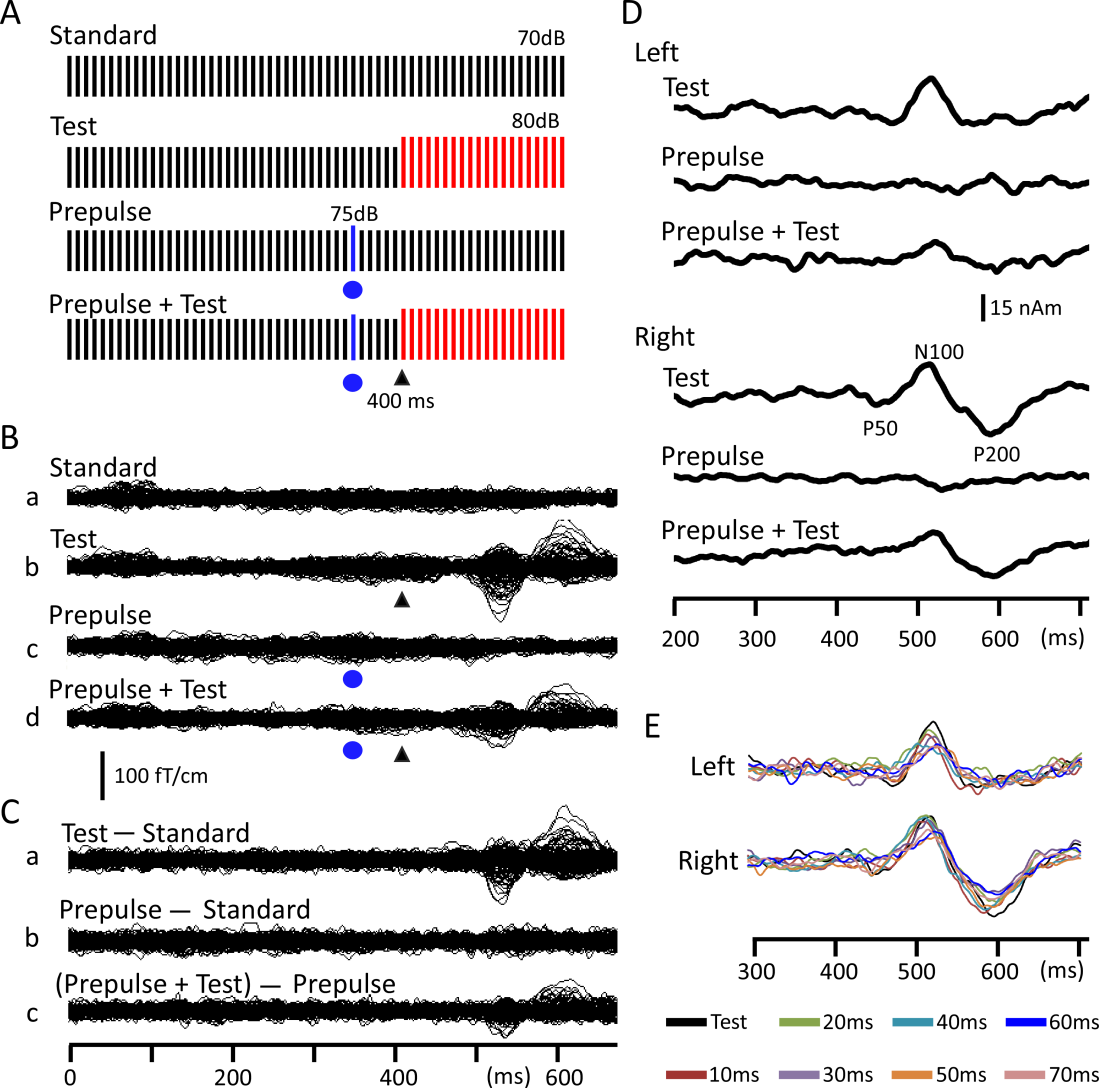
Change-related cortical response and its inhibition by a weak prepulse. (A) Sound stimuli consisted of a train of 1-ms clicks at 100 Hz in repetitive frequency and 70 dB SPL in sound pressure. An abrupt increase of 10 dB in sound pressure was used to evoke the Test response, while that for the prepulse (5 dB) was used to inhibit the Test response. Each bar indicates a single click. (B) An example of prepulse inhibition of the change-related auditory response by a prepulse presented 60 ms before the Test stimulus in a representative subject. Recorded MEG waveforms in the Pre run of the Placebo session for the Standard (Ba), Test (Bb), Prepulse (Bc), and Prepulse + Test (Bd) stimuli are shown. The Test alone MEG response (Ca) was obtained by subtracting the waveform for the Standard stimulus (Ba) from that for the Test stimulus (Bb). The Prepulse alone response (Cb) was obtained by subtracting the waveform for the Prepulse stimulus (Bc) from that for the Standard (Ba). The Prepulse + Test response (Cc) was obtained by subtracting the waveform for the Prepulse stimulus (Bc) from that for the Test + Prepulse stimulus (Bd). (D) Time course of the source strength of dipoles obtained from MEG waveforms in C. (E) The source strength waveforms for all eight PTI conditions in this run. Filled blue circles and black arrow heads indicate the presentation of the prepulse and test stimulus, respectively.

Repeats of a 1-ms sine wave click at 100 Hz [10] were used. There were four types of sound stimuli (Fig 1A): repeats of the same click at 70 dB SPL in sound pressure (Standard), repeats of standard clicks followed by 20 clicks of 80 dB (Test), the Test preceded by a click of 75 dB (Prepulse + Test), and the Standard with a Prepulse (Prepulse). Prepulses were presented 10, 20, 30, 40, 50, 60, or 70 ms before the Test presented at 400 ms in the click train. Therefore, there were 16 stimuli: Standard, Test, Prepulse alone with a prepulse at 330–390 ms, and Prepulse + Test with a prepulse at 330–390 ms. Sixteen stimuli were presented randomly at an even probability at a trial-trial interval of 1000 ms. A total of 120–125 artifact-free epochs were averaged for each stimulus. Sound stimuli were presented binaurally through ear pieces (E-A-Rtone 3A, Aero Company, Indianapolis, IN).

### MEG recordings

Magnetic signals were recorded using a 306-channel whole-head type MEG system (Vector-view, ELEKTA Neuromag, Helsinki, Finland), which comprised 102 identical triple sensor elements. Each sensor element consisted of two orthogonal planar gradiometers and one magnetometer coupled to a multi-superconducting quantum interference device (SQUID) and, thus, provided 3 independent measurements of the magnetic fields. In the present study, we analyzed MEG signals recorded from 204 planar-type gradiometers. These planar gradiometers are sufficiently powerful to detect the largest signal just over local cerebral sources. Signals were recorded with a bandpass filter of 0.1–330 Hz and digitized at 1000 Hz. An analysis was conducted from 100 ms before to 300 ms after the onset of the Test. Epochs with MEG signals larger than 2.7 pT / cm were rejected from averaging. Subjects sat in a chair and watched a silent movie on a screen 1.5 m in front of them throughout the experiments.

### Procedures

All subjects participated in three experimental sessions: Placebo, Diazepam, and Baclofen, on different days spaced by more than one week. The order was randomized among subjects. In each session, there were two MEG measurement runs: Pre (baseline) and Post. At the beginning of the MEG recording, brain activity during a 2-min eye-closed rest period was recorded in order to assess the dominant alpha frequency of background activity. Thereafter, we recorded auditory evoked magnetic fields for the baseline run (Pre). After the Pre run, subjects were blindly administered a placebo, diazepam (5 mg), or baclofen (10 mg) tablet. The dosage of diazepam and baclofen was approximately one-third of the standard daily dosage. One hour (Placebo and Diazepam)[15,16] or 2.5 hours (Baclofen)[17] after drug administration, 2-min background activity was recorded again and the Post run was then conducted. At the end of each run, subjects reported their sleepiness during the run using the Stanford Sleepiness Scale questionnaire [18]. This was done because the two drugs often cause sleepiness but we did not know the effect of vigilance on PPI. The time needed to complete a run was approximately 40 min.

### Analysis

Recorded MEG waveforms were subjected to band-pass filtering of 1–75 Hz and analyzed as previously reported [9–11]. In brief, the Test-alone response (Panel C, Part a in Fig 1) was obtained by subtracting the waveform for the Standard (Ba) from that for the Test alone stimulus (Bb). Similarly, the Prepulse + Test response (Cc) was obtained by subtracting the waveform for the Prepulse alone stimulus (Bc) from that for the Prepulse + Test stimulus (Bd). Using the subtracted Test-alone response waveform for the Pre run, an equivalent current dipole for the magnetic component at approximately 130 ms, Change-N1m, was estimated at the peak latency for each hemisphere using BESA (NeuroScan, Mclean, VA). A dipole model was obtained for the Test-alone response of all three sessions: Placebo, Diazepam, and Baclofen, and the orientation and location of the dipole for the Test-alone response were then averaged across three sessions. The dipole model obtained was applied to all subtracted MEG waveforms (Fig 1C) of all sessions, and the source strength waveform was used to measure the amplitude of Change-N1m. Fig 1D shows the source strength waveforms of the Pre run of the Placebo session in a representative subject. Fig 1E shows the superimposed test-evoked source strength waveforms of all eight PTI conditions.

The peak amplitude of Change-N1m was measured between the peak in Change-N1m within the period of 100–200 ms and the peak in the polarity-reversed earlier component within 50–100 ms after the change onset to minimize issues associated with a baseline shift [9,14]. The percent inhibition of the Change-N1m amplitude by the prepulse (%PPI) was defined as (Test alone response–(Prepulse + Test response)) / Test alone response * 100. The Change-N1m amplitude or degree of inhibition was compared among Test-evoked responses using a three-way repeated measures ANOVA with Prepulse, Hemisphere, and Drug as the independent variables. The effects of drugs on PPI were compared between placebo and diazepam, and between placebo and baclofen. Changes in %PPI between Pre and Post runs were calculated for each PTI of each drug, and the difference was compared using a three-way ANOVA (Hemisphere x Drug x PTI) followed by paired *t*-tests corrected with Bonferroni adjustments for multiple comparisons. In addition, paired comparisons for each PTI between placebo and diazepam and between placebo and baclofen were conducted using the bootstrapping resampling method (2000 times). We calculated confidence intervals of resampled data and tested whether they covered zero. IBM SPSS Statistics (version 21) was used for statistical analyses.

In order to assess the dominant alpha rhythm, a stable period of 2 min was selected and subjected to FFT. Twenty symmetric sensors in the occipital area were chosen and the peak frequency of background activity was averaged across 20 sensors [19]. The dominant alpha rhythm of the background activity and sleepiness score were compared between Pre and Post runs using paired *t*-tests. All statistical analyses were performed at the 0.05 level of significance. Data are expressed as the mean ± standard deviation (SD).

## Results

### Baseline responses

**Fig 2:**
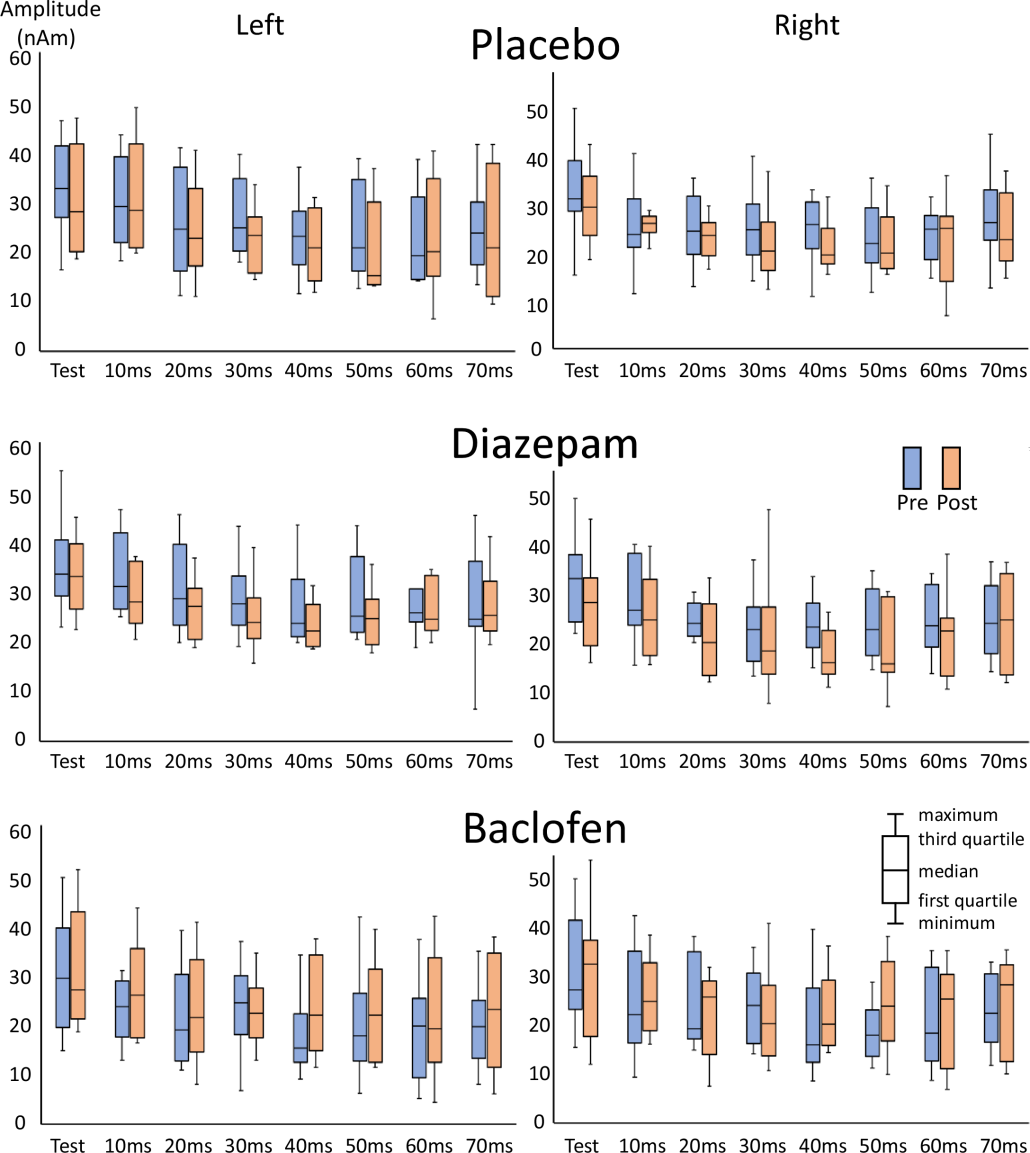
Amplitudes of the change-related response. The mean amplitude of the test response across subjects for all PTI conditions is shown in box plots.

The test stimulus, an abrupt increase of 10 dB from the background (Fig 1A), evoked a clear change-related response (Fig 1B). As in previous studies [9,10,14], the generator of the N100 component (Fig 1D) was estimated to be located in and around the superior temporal gyrus. The peak amplitude of each condition is shown in Fig 2 and Table 1. Among the three baseline (Pre) runs before drug administration, the results of a three-way ANOVA (Hemisphere x Drug x PTI) showed that the peak amplitude was significantly different among PTIs (F_7,63_ = 17.0, p = 2.1 x 10^−12^), but not among drugs (F_2,18_ = 2.31, p = 0.13) or between hemispheres (F_1,9_ = 0.02, p = 0.89). Post hoc tests showed that all prepulses, except 10-ms PTI, significantly suppressed the test response (P < 0.002), confirming the strong effect of the prepulse. The greatest suppression was obtained by 40-ms PTI. Fig 1D shows an example of waveforms for the Test alone, Prepulse alone, and Prepulse + Test responses with a prepulse presented 60 ms before the test stimulus.

**Table 1:** The peak amplitude of the change-related auditory response.

	Left				Right			
Placebo	Pre		Post		Pre		Post	
Prepulse	Amplitude	%PPI	Amplitude	%PPI	Amplitude	%PPI	Amplitude	%PPI
Test alone	33.3(9.3)		30.0(10.7)		31.4(8.9)		28.7(7.2)	
10ms	29.9(8.9)	9.4(12.4)	30.4(10.3)	-2.9(16.3)	24.7(8.2)	21.8(11.1)	25.4(3.7)	8.5(17.0)
20ms	25.3(10.4)	25.4(14.8)	24.2(9.2)	19.2(12.4)	23.7(7.5)	24.3(12.5)	23.3(6.3)	18.0(12.7)
30ms	26.6(7.8)	18.0(17.7)	22.5(6.3)	22.4(13.8)	24.4(7.6)	21.6(12.1)	20.7(6.8)	28.2(11.9)
40ms	23.0(7.4)	28.8(19.3)	21.2(7.7)	28.7(13.5)	24.1(6.6)	22.3(14.7)	20.4(5.0)	25.5(24.0)
50ms	23.7(9.3)	27.8(19.9)	20.5(8.9)	32.1(11.3)	22.3(7.2)	29.1(8.4)	21.5(6.2)	23.5(18.5)
60ms	22.7(9.2)	30.6(21.8)	23.3(10.9)	22.8(23.9)	22.6(5.4)	25.4(16.5)	21.0(8.6)	25.9(30.0)
70ms	24.5(8.8)	23.9(24.2)	24.0(12.8)	23.1(23.5)	26.8(9.2)	15.2(11.6)	24.0(7.7)	14.0(26.3)
**Diazepam**								
Test alone	30.9(11.0)		28.9(8.7)		32.3(8.5)		27.5(9.0)	
10ms	29.1(9.0)	2.5(20.4)	23.8(7.3)	16.3(16.1)	28.9(7.9)	6.5(30.8)	25.6(8.1)	5.6(11.7)
20ms	26.0(10.8)	16.8(13.4)	21.2(6.7)	26.8(6.0)	26.4(7.9)	16.3(18.6)	21.4(7.6)	22.0(11.6)
30ms	23.8(9.3)	23.0(10.9)	19.2(7.7)	33.1(18.0)	22.7(7.3)	29.4(15.2)	21.5(11.9)	25.2(19.3)
40ms	20.9(8.7)	32.3(11.3)	17.4(5.5)	38.5(12.0)	23.5(5.8)	26.0(13.4)	18.8(8.2)	30.8(16.5)
50ms	23.4(10.2)	24.4(16.0)	18.9(7.3)	33.2(19.8)	23.7(7.2)	26.0(15.2)	19.6(8.3)	28.3(21.6)
60ms	22.5(8.9)	25.7(19.8)	21.2(6.7)	24.1(21.5)	24.8(6.9)	21.1(19.9)	21.4(7.9)	22.2(13.8)
70ms	22.8(13.4)	30.6(32.2)	22.0 (8.0)	23.1(16.3)	24.7(7.8)	22.6(19.9)	24.0(9.2)	13.5(17.5)
**Baclofen**								
Test alone	29.4(11.2)		31.0(11.4)		30.9(11.3)		30.3(12.1)	
10ms	24.9(9.9)	12.2(23.5)	27.0(9.2)	11.6(14.1)	23.4(9.5)	23.5(19.1)	25.2(7.5)	10.6(22.6)
20ms	21.9(10.3)	26.7(14.0)	23.0(10.7)	26.4(22.5)	23.7(9.1)	19.9(26.2)	21.9(8.2)	26.0(15.8)
30ms	23.6(9.1)	19.8(15.5)	22.8(6.7)	23.7(14.8)	23.4(7.5)	21.4(18.1)	21.5(9.5)	27.3(17.2)
40ms	18.5(9.7)	37.4(16.3)	23.0(9.5)	26.5(10.7)	18.9(9.3)	39.8(12.3)	22.2(7.4)	21.9(20.2)
50ms	20.2(10.9)	31.9(18.8)	22.8(9.4)	27.1(13.0)	17.9(5.2)	39.1(14.7)	23.8(9.5)	19.8(16.6)
60ms	19.1(9.9)	36.2(19.8)	21.5(12.2)	33.2(24.9)	19.7(8.3)	36.8(9.9)	21.5(10.2)	30.1(16.7)
70ms	19.8(8.5)	32.3(15.6)	22.4(11.7)	29.0(26.4)	21.8(6.7)	26.7(15.0)	23.3(10.0)	20.5(25.9)

Amplitudes (nAm) are shown as the mean (SD)

When percent inhibition (%PPI) was compared among the three Pre runs, the results obtained were similar; PTI significantly affected the degree of suppression (F_6,54_ = 5.27, p = 2.5 x 10^−4^), whereas Hemisphere (p = 0.90) and Drug (p = 0.13) did not. Bonferroni’s post hoc tests showed that %PPI was significantly greater for 40-ms PTI than for 10-ms (p = 0.008) and 30-ms (P = 0.015) PTIs.

### Effects of drugs

**Fig 3:**
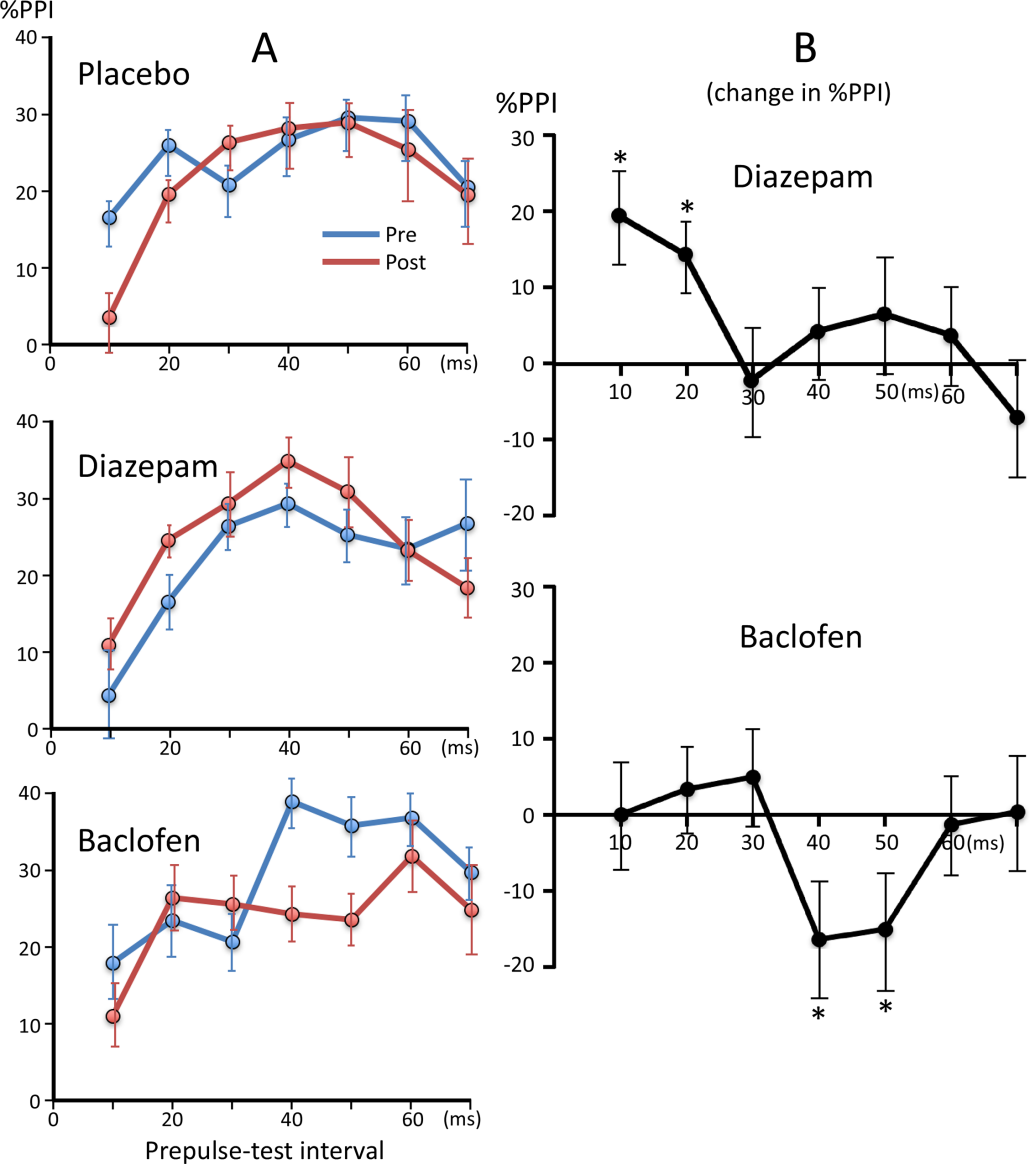
Percent inhibition at each prepulse-test interval before and after drug administration. The mean %PPI across subjects are shown. (A) Plots of %PPI against PTI before and after each drug administration. (B) Changes in %PPI following diazepam and baclofen administration after normalization by subtracting the values of the placebo session. *, p < 0.05 significantly different from the Placebo (paired *t*-test uncorrected for multiple comparisons). Error bars show ± the standard error.

The results of paired *t*-tests showed that none of the three drugs significantly altered the amplitude of the test response for both hemispheres (p > 0.05, uncorrected for multiple comparisons). Fig 3 shows plots of %PPI against PTI before and after the drug treatment. In order to analyze the effects of drugs, we calculated differences in %PPI between Pre and Post runs and the difference was then subjected to a three-way ANOVA (Hemisphere, Drug, and PTI). When the difference in %PPI was compared between placebo and diazepam, the results of the ANOVA showed that none of the factors significantly affected %PPI (p > 0.13), while the Drug x PTI interaction was significant (F_6,54_ = 2.37, p = 0.042). The difference in %PPI for each PTI was compared between placebo and diazepam using paired *t*-tests with Bonferroni adjustments for multiple comparisons. The results obtained showed that the administration of diazepam significantly enhanced the suppression of 10- (p = 0.035) and 20-ms (p = 0.042) PTIs. Fig 3B shows changes in %PPI after diazepam, from which changes after the placebo were subtracted. In the comparison between placebo and baclofen, the Drug x PTI interaction was significant (F_6,54_ = 2.93, p = 0.015). As shown in Fig 3B, baclofen decreased the %PPI for 40- and 50-ms PTIs. Although uncorrected *t*-tests showed that the difference was significant for both conditions (p = 0.023 and 0.026, respectively), it was not after the Bonferroni correction.

As shown in Fig 2, fluctuations were observed in baseline PPI among sessions, which may affect statistical evaluations of drug effects. Therefore, we conducted an additional analysis for these four PTIs. The effects of drugs were compared between placebo and diazepam, and between placebo and baclofen using the bootstrapping resampling (2000 times) method. The results obtained showed a significant effect by diazepam at 10 (p < 0.00001) and 20 ms (p < 0.001), and by baclofen at 40 and 50 ms (p < 0.05).

### Sleepiness and background alpha rhythm

The sleepiness score was similar between Pre and Post runs for placebo (2.2 ± 1.3 and 2.1 ± 1.1, respectively). Although diazepam (2.0 ± 1.1 and 2.9 ± 1.4) and baclofen (1.5 ± 0.8 and 2.0 ± 1.2) increased the score for the Post run, the difference was not significant (p = 0.12, uncorrected for multiple comparisons). While the background alpha rhythm was slightly slower for the Post run of all drugs (10.1 ± 1.1 and 9.8 ± 0.9 for placebo; 10.0 ± 0.8 and 10.0 ± 1.1 for diazepam; 10.3 ± 1.1 and 10.1 ± 1.0 for Baclofen), the difference was not significant (p = 0.28, 0.76, and 0.07, respectively; uncorrected for multiple comparisons).

## Discussion

The present study confirmed the PPI of change-related cortical responses by a weak change stimulus that itself evoked no or only a weak response (Fig 1B). Suppression by such a weak leading stimulus indicates that this suppression is not due to passive events such as fatigue, but rather due to active inhibitory processes. The present study confirmed this, showing that the GABA system participated in PPI. By manipulating the PTI, a previous study suggested that at least three different mechanisms are involved in the PPI of the change-related cortical response showing peaks at PTIs of approximately 20–30, 50–60, and 500–600 ms [11]. The present study pharmacologically separated at least two mechanisms for short-latency inhibition.

The results of the present study showed enhanced PPI at 10- and 20-ms PTIs following the administration of diazepam, suggesting that GABA_A_ receptors are involved in short-latency inhibition. Although PPI at the 10-ms PTI did not significantly suppressed the test response in the present study, the difference was significant in our previous study [11], suggesting that although the inhibitory effects of prepulses with short PTIs were weak, their actions became obvious in the presence of diazepam. However, the significant effect of diazepam at the 10-ms PTI depended on slightly decreased PPI for placebo and slightly increased PPI for diazepam. The difference was not significant without subtraction of the placebo effects. The GABA_A_ receptor is ionotropic and when it is activated by its endogenous ligand GABA, the chloride ion-selective pore opens. This causes hyperpolarization of the membrane and, thus, decreases temporally overlapping EPSPs in target cells. Besides the active site for GABA, the GABA receptor has a number of other bindings sites, one of which, the benzodiazepine site, is the target of diazepam [20]. Such an allosteric modulator indirectly influences the effects of GABA. Diazepam augments effects through the GABA_A_ receptor by increasing the frequency of channel openings [21]. In whole-cell recording studies, GABA_A_-mediated early IPSPs or IPSCs were enhanced by the bath application of diazepam [22–24].

Since the time course of PPI in the present study is considered to reflect that of IPSPs being negligibly confounded by EPSPs produced by a prepulse [11], the present results indicate that early IPSPs at 10–20 ms were induced through GABA_A_ receptors. This is consistent with the peak latency of the early GABA_A_-mediated IPSPs reported in whole-cell studies of approximately 10–30 ms [25–28]. Although several types of interneurons elicit fast IPSPs [28], we considered parvalbumin-positive (PV) cells to be a possible candidate for early inhibition [11]. PV or fast spiking (FS) interneurons, the largest subclass of interneurons [29,30], play a role in fast inhibition [31]. While the present study was unable to identify the cell type, the results obtained indicate that at least one interneuron type sensitive to benzodiazepine participated in the present PPI.

Baclofen slightly suppressed PPI at PTIs of 40–50 ms, suggesting the involvement of GABA_B_ receptors [32] in the present PPI. However, unlike diazepam, the interpretation of results is not straightforward. GABA_B_ receptors are metabotropic and induce late inhibition through G-protein activation [33]. Baclofen is an agonist of GABA_B_ receptors and induces late IPSPs in target cells. However, it has also been shown to block GABA release via presynaptic autoreceptors [34–37] (for a review, see Misgeld et al. [38]). In addition, GABA_B_ receptors are located on glutamate fiber terminals [39]. Therefore, its overall effects on a circuit depend on which receptors are predominantly activated. In the present study, no significant differences were observed in the amplitude of the test response before and after the administration of baclofen, suggesting that its actions on the presynaptic GABA_B_ receptors of glutamatergic cells did not play a major role. Baclofen appears to have suppressed GABA release via autoreceptors on the axon terminals of GABAergic neurons in the present study. Therefore, the present results suggest that GABA_B_-mediated inhibition peaked at 40–50 ms, which is consistent with GABA_B_-mediated IPSPs following GABA_A_-mediated fast IPSPs. Although the peak latency of GABA_B_-mediated IPSPs varied in whole-cell recording studies, it was always markedly longer than that of early IPSPs [26,27,40,41]. A previous study using the microapplication of glutamate to rat cortical slices showed late IPSPs sensitive to 2-hydroxysaclofen, a blocker of GABA_B_ receptors, peaking at 45 ms [42], which is consistent with the present results.

In the present study, 10 mg of baclofen was used. Since baclofen does not readily penetrate the blood brain barrier [43], its brain concentration in the present study was expected to be very low. However, presynaptic GABA_B_ receptors are more sensitive to GABA than postsynaptic receptors [44]. Baclofen inhibits GABA release from axon terminals at a low concentration at which it exerts no postsynaptic effect. Scholfield et al. showed in olfactory cortex slice preparations from guinea pigs that the bath application of 0.2 μM baclofen almost completely abolished IPSCs evoked by a stimulation of the lateral olfactory tract [45]. Guetg et al. [46] examined the roles of the GABA_B_ receptor subtypes, GABA_B(1a, 2)_ and GABA_B(1b, 2)_, in the inhibition of transmitter release and demonstrated that 0.1 μM baclofen was exclusively effective for GABA_B(1a, 2)_ receptors that were preferentially distributed on presynaptic sites. Since the cerebrospinal fluid concentration of baclofen was expected to be 0.07–0.13 μM in the present study [43,47], it is possible that baclofen under the present experimental conditions acted dominantly on presynaptic GABA_B_ receptors and the inhibition of GABA release resulted in decreased PPI. Regarding GABA_A_-mediated inhibition, the present results showed that baclofen did not affect PPI at PTIs of 10 and 20 ms, whereas diazepam significantly increased inhibition, suggesting that GABA_A_-induced inhibition was not affected by presynaptic GABA_B_ receptors. In patch clamp studies, these effects of baclofen were shown to be stronger for GAGA_B_-induced late IPSPs than GABA_A_-induced early IPSPs [35,41]. Connors et al. [41] reported that baclofen abolished late IPSPs, while leaving early IPSPs almost intact.

Therefore, the present results suggest that baclofen reduced GABA-mediated inhibition, which may be associated with seizures after the intrathecal application of baclofen [48] because hypofunction of the GABA_B_ receptor is considered to cause epilepsy [49]. In support of this, Dugladze et al. [37] demonstrated that baclofen at a low concentration induced the hyperexcitability of hippocampal neurons in epileptic mice, and this was due to the enhanced inhibition of GABA release via presynaptic GABA_B_ receptors.

GABA functions are considered to be related to susceptibility to epilepsy; many anticonvulsants act on GABA receptors, GABA receptor antagonists or negative allosteric modulators act as a convulsant, GABA receptor knockout animals develop epilepsy, many animal models of epilepsy show abnormalities in GABA functions, and GABA elicits the hyperpolarization of target neurons, making them fire less [38,50,51]. Therefore, the present measure may serve as a clinical test for susceptibility to epilepsy.

Besides epilepsy, GABA is considered to play a role in the pathophysiology of many diseases such as schizophrenia [1–4] and autism spectrum disorder (ASD)[5]. Due to the lack of appropriate methods for functional measurements of the GABA system, the present method appears to be a useful tool for understanding the pathophysiology of these diseases and estimating inhibitory functions in individuals. However, it currently remains unclear whether inhibition in a specific circuit in the present study reflects the fundamental function of the GABA system of the individual. In whole-cell recordings, the sequence of early IPSPs mediated by GABA_A_ receptors and late IPSPs mediated by GABA_B_ receptors is common across species [41], including humans [27], and brain areas [52,53]. Furthermore, FS or PV interneurons are known to be densely connected to nearby pyramidal cells in a non-selective manner across cortical areas and layers, suggesting their role in unspecific inhibition [52]. Similar unspecific dense connections to neighboring pyramidal cells, a blanket of inhibition, have also been reported for the interneurons responsible for long-latency inhibition [54]. Therefore, inhibition in the change-driven circuit of the auditory cortex in the present study may represent a basic inhibitory mechanism and the present method serves as a tool for translational studies.

ASD, for example, is considered to be associated with alterations in GABAergic signaling [5,55]. The high prevalence of epilepsy in ASD [56] further supports this idea and common mechanisms underlying both disorders have been investigated [57,58]. *In vitro* analyses of the post-mortem brain tissues of ASD patients showed reductions in GABA receptor density or glutamic acid decarboxylase (GAD) in various brain areas including the anterior cingulate cortex, fusiform gyrus, hippocampus, parietal cortex, and cerebellum [55]. *In vivo* studies using positron emission tomography (PET), single photon emission computed tomography (SPECT), and magnetic resonance spectroscopy (MRS) showed reduced levels of GABA receptors or GABA concentrations in the frontal lobe, perisylvian region, superior and medial frontal cortex, fronto-temporal cortex, auditory cortex, motor cortex, anterior cingulate cortex, and limbic areas (for a review, see Cellot & Cherubini [55]; Dickinson et al. [59]). These findings indicate alterations in the GABA system in various brain areas in ASD, thereby suggesting regional non-specific fundamental changes in the GABA system. However, the function of the inhibitory system in ASD is unknown. In animal models of ASD, PV interneurons are reduced in the neocortex across multiple models [60], suggesting common PV-circuit disruption across animals showing ASD behavioral phenotypes. In animals, unlike humans, IPSCs produced through GABA receptors may be observed using whole-cell recording techniques [61,62]. For example, Lo et al. [63] showed markedly reduced GABA_A_-mediated IPCSs in the barrel cortex of ASD model mice, confirming deficits in the GABA system. However, *in vivo* recordings of IPSPs are challenging and, to the best of our knowledge, this has not yet been conducted on ASD model animals. Measurements of the function of the GABA system *in vivo* are difficult in humans and animals. The present measure is easily performed using electroencephalograms [12] and, thus, may also be applied to animals, which enables comparisons of data between animals and humans. Similar measurements in animals are needed in the future.

### Limitations

The test response in the present study was evoked by a sound feature change in a continuous click train. Since neurons in the auditory cortex easily adapt to a repetitive sound stimulus [64,65], the generation of change-related activity appears to be based on disinhibition of the change-detecting system. Although the generation mechanism of change-related brain activity is unclear, inhibitory circuits may be involved. A recent computational study on stimulus-specific adaptation by Nelken and Yarden [66] supported this view. Our results showed that diazepam and baclofen did not significantly alter the test response, suggesting that this was not the main mechanism responsible for the present results showing the significant impacts of these drugs on the test response. However, these drugs may have altered brain sensitivity to a change and, thus, affected the results obtained.

The systemic administration of drugs was problematic. Although the sleepiness score did not significantly differ among the three sessions, diazepam and baclofen caused slight sleepiness in some subjects. Since we did not know the effects of vigilance on PPI, the possibility of this effect on the results obtained cannot be denied. Furthermore, the anxiolytic effects of diazepam may have affected the present results. This may be problematic when these effects differ among inhibitory mechanisms.

To understand whether this method can be applied to pathological conditions, further studies are necessary. The baseline PPI showed fluctuations among the three sessions, which is important if the present method is used as a clinical test. For example, the present results for the PPI with the 10-ms PTI showed reduced inhibition after placebo administration and a slight increase after diazepam. Without normalization procedures, the effect of diazepam was not significant, which may hamper interpretation of the results. Therefore, the consistency of measurement across sessions is important. This was due, at least in part, to the low S/N ratio of the evoked response. Since there were eight conditions in one run, we had to use a minimum number of trials for averaging. The present results revealed some key PTIs for testing, and, thus, we may reduce the number of conditions. Future studies with fewer conditions, but with a greater amount of averaging will confirm if more stable data may be obtained. For applying to patients, test-retest reliability should be tested using electroencephalograms. Because clinical importance of PPI of startle reflexes is well known [67], research on the relationship between the conventional PPI and present PPI is also necessary. Since the sensory cortex is outside the conventional PPI pathway, the present results may suggest that the prepulse-induced suppression is not specific to startle pathways but represents a non-specific mechanism of sensory processing [11].
